# Editorial: Exploring novel experimental systems to study the mechanistic basis of fungal infections

**DOI:** 10.3389/ffunb.2023.1195041

**Published:** 2023-04-14

**Authors:** Sara Gago, Mark S. Gresnigt, Teresa Zelante

**Affiliations:** ^1^ Manchester Fungal Infection Group, The University of Manchester, Manchester, United Kingdom; ^2^ Junior Research Group Adaptive Pathogenicity Strategies, Leibniz Institute for Natural Product Research and Infection Biology - Hans Knöll Institute, Jena, Germany; ^3^ Department of Medicine and Surgery, Pathology Section, University of Perugia, Perugia, Italy

**Keywords:** animal models, fungal disease, *in vitro* infection model, *Aspergillus*, sporotrichosis, *coccidioidomycosis*, *Cryptococcus*, *Caenorhabditis elegans*

## Introduction

Invasive fungal diseases are a significant cause of morbidity and mortality worldwide. In the last years the increment of at-risk populations for fungal diseases has been linked with new clinical procedures including immunosuppresive therapy, use of invasive devices, transplantation, but also autoimmunity, diabetes, severe primary infections, cancer, or chronic lung diseases. The diagnosis of invasive fungal diseases is difficult as signs and symptoms are unspecific, thus delaying the start of antifungal therapy. Moreover, resistance to antifungals is emerging, and naturally resistant species such as *Candida auris* are on the rise. In addition, there are new risk factors for fungal disease such as the use of targeted biologics inhibiting important players in antifungal host defense, including IL-17A, IL-17RA, IL-12p40, Tumor necrosis factor α (TNFα). Even though during the last few decades there has been a significant advance in our understanding the mechanistic basis of fungal disease, research in this field has been hampered by difficulties in disease modelling.

In this Research Topic we aimed to explore how the use of novel experimental systems can be used to answer important questions about the mechanistic basis of fungal disease. These approaches can help us to better understand the timeline of infection from development to progression, identify critical disease biomarkers that could improve diagnosis, and discover those genes or pathways that could serve as novel therapeutic targets. For these reasons, in this Research Topic we wanted to integrate new methodological approaches developed by early career researchers with a view to improving our understanding in fungal pathogenesis. The application of these technologies to answer different research questions would allow us to improve the diagnosis and treatment of patients with these deadly infections. Here we included research articles and reviews focused on immunology, fungal biology, host-pathogen interactions, fungal pathogenicity mechanisms, and unknown fungal-microbiome connections.

## Key highlights of published articles

Three original research articles, one brief research article, one review and this editorial are included in this Research Topic.

The Research Topic begins with an original research article by Powell et al. focused on the role of Tumor necrosis factor α (TNFα) in *Coccidioides* infection. Coccidiomycosis is an endemic mycosis in the Americas. Clinical management of these patients usually requires long-term antifungal treatment and the use of immunotherapy, as proposed in this article, is of high interest. Nevertheless, the study of *Coccidioides* infection is complicated by its BSL3 status and the fact that it is difficult to generate infectious spores. The authors used a novel mouse model to dissect the importance TNFα-mediated response in coccidiomycosis. In their model they suggested that the use of an *in vivo* model of ‘static infection state” would recapitulate the phenotype of patients with good resistance to *Coccidiodides* infections (Shubitz et al., 2021). Using this model, they have shown that B6D2F1 mice are resistant to lethal infection with Cp1038 strain. However, mice were unable to control the infection when TNFα was neutralized. Powell et al. elegantly showed that anti-TNFα, treatment increases susceptibility of mice to *Coccidioides* infection. This significant impairment of mice to overcome *Coccidioides* infection happens even though treatment is stopped and provides a theoretical framework to investigate how disruption of TNFα signalling facilitates coccidiomycosis disease progression. These findings strongly support human studies where genetic variants in Dectin-1 in patients associated with impaired TNFα production and are linked with increased risk for development of disseminated disease (Hsu et al., 2022).

The second original research article published in the Research topic is focused on the development of a novel mouse model of *Cryptococcus neoformans* infection, where persistent infection due to latency was achieved. Ding et al. used clinical isolates from HIV patients with cryptococcal meningitis. Mice infected with clinical *C. neoformans* isolates demonstrated persistent, stable infections with low fungal burden, for more than 90 days-post infection. These mice also exhibited weight gain and no clinical symptoms of disease, with no inflammation, emphasizing how this new mouse model system can facilitate studies focused on understanding host immune responses that keep *C. neoformans* infection under control. Mice with latent infections were subsequently depleted of Th1 cells, leading to the development of lethal disseminated infection. Thereby this model provided a mechanistic basic support to the essential role of IFN-γ to control *C. neoformans* infections in patients with HIV (Lee et al., 1996).

Vargas-Macías et al. reviewed available models of sporotrichosis, a global distributed subcutaneous mycosis that affects mammals, including humans. Sporotrichosis is acquired through direct inoculation of conidia on vegetation surfaces or by zoonotic transmission. There are different species of the *Sporothrix* genus that can cause diseases, but all species differ in their virulence. In this review, Vargas-Macías et al. summarized the utility of *in vitro*, *in vivo*, *ex vivo*, and invertebrate models to study the pathophysiology of Sporotrichosis. The authors highlighted the strength of combining different experimental approaches to study host-pathogen interactions in this context. Readers of this review can profit from a unique opportunity to gain an in-depth understanding of current experimental systems, as well as their advantages and disadvantages to study host-pathogen interactions and pharmacological interventions.

The third research article published in the Research topic is focused on aspergillosis. Silva-Ferreira et al. demonstrated the utility of a mouse model of *Aspergillus fumigatus* airway infection in myeloid-restricted HIF-1α knock-out (*mHif1*α^-/-^) mice. In this model, *A. fumigatus* was delivered in coated-beads that facilitate the formation granulomas in infected lungs thus mimicking aspects of the pathohysiology of chronic pulmonary aspergillosis. While HIF-1α-deficient mice showed smaller granulomas, these mice suffered from uncontrolled fungal growth with enhanced neutrophil cell death. This work provides further important insights in the connection between the hypoxia sensor HIF-1α and protective antifungal immunity. Moreover, this model is also a crucial step forward towards the modelling of chronic fungal diseases and opens future avenues for mechanistic studies.

The last original research article of the topic is focused on modelling of cryptococcal meningitis by using a *Caenorhabditis elegans* infection model. The *C. elegans* model of neurodegeneration presented in this article displays several advantages compared to other models of disease mainly due to their simple anatomy, transparency, and brief lifespan (Tang et al., 2005). Using this model Kitisin et al. demonstrated that neurodegeneration occurred as a direct consequence of the cryptococcal infections. Thus, *C. elegans* infection model of cryptococcal-induced neurodegeneration represents a valid alternative to mouse models for evaluation of novel therapeutics to prevent neuronal damage during fungal infections.

## Conclusion

This Research Topic clearly shows the need to develop new physiological models of fungal diseases and underscores the urgent need for the development of models that better recapitulate pathophysiological mechanisms. There is a need for models that can be used to accurately study infections that are organ specific. The development of infection models to study fungal diseases is limited by the intrinsic capacity of fungi to adapt in the host environments, where they may induce latency, granulomas, static infection states, neurodegeneration, and the huge variety of susceptible hosts. Future research should aim to standardize infection models that better recapitulate the complexity of the host-pathogen interface as shown for a small selection of fungal pathogens by the authors contributing to this Research Topic.

## Author contributions

SG, TZ, and MG conceived the Research Topic and edited the manuscripts. TZ wrote the first draft of the editorial. All authors read and provided significant inputs into all drafts of the editorial, agreed to be accountable for all aspects of the work and approved the final draft of the editorial for publication.
